# Exploiting Substrate Specificities of 6-*O*-Sulfotransferases to Enzymatically Synthesize Keratan Sulfate Oligosaccharides

**DOI:** 10.1021/jacsau.3c00488

**Published:** 2023-10-13

**Authors:** Yunfei Wu, Gaël M. Vos, Chin Huang, Digantkumar Chapla, Anne L. M. Kimpel, Kelley W. Moremen, Robert P. de Vries, Geert-Jan Boons

**Affiliations:** † Department of Chemical Biology and Drug Discovery, Utrecht Institute for Pharmaceutical Sciences, Utrecht University, Universiteitsweg 99, Utrecht 3584 CG, The Netherlands; ‡ Complex Carbohydrate Research Center, University of Georgia, 315 Riverbend Road, Athens, Georgia 30602, United States; § Department of Biochemistry, University of Georgia, Athens, Georgia 30602, United States; ∥ Bijvoet Center for Biomolecular Research, Utrecht University, Padualaan 8, Utrecht 3584 CH, The Netherlands; ⊥ Department of Chemistry, University of Georgia, Athens, Georgia 30602, United States

**Keywords:** chemoenzymatic synthesis, keratan sulfate, glycosyltransferases, influenza A, Siglec

## Abstract

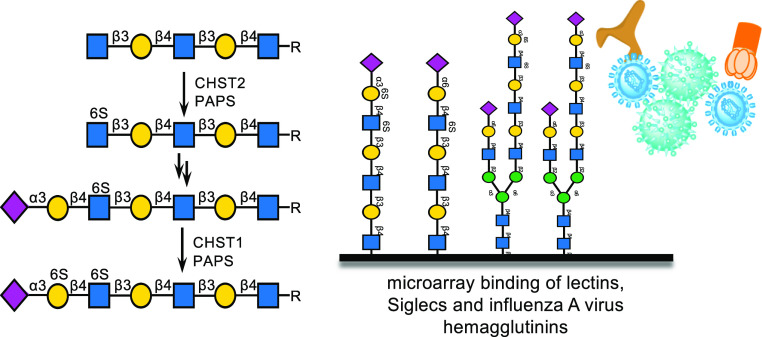

Keratan sulfate (KS) is a glycosaminoglycan that is widely expressed in the extracellular matrix of various tissue types, where it is involved in many biological processes. Herein, we describe a chemo-enzymatic approach to preparing well-defined KS oligosaccharides by exploiting the known and newly discovered substrate specificities of relevant sulfotransferases. The premise of the approach is that recombinant GlcNAc-6-*O*-sulfotransferases (CHST2) only sulfate terminal GlcNAc moieties to give GlcNAc6S that can be galactosylated by B4GalT4. Furthermore, CHST1 can modify the internal galactosides of a poly-LacNAc chain; however, it was found that a GlcNAc6S residue greatly increases the reactivity of CHST1 of a neighboring and internal galactoside. The presence of a 2,3-linked sialoside further modulates the site of modification by CHST1, and a galactoside flanked by 2,3-Neu5Ac and GlcNAc6S is preferentially sulfated over the other Gal residues. The substrate specificities of CHST1 and 2 were exploited to prepare a panel of KS oligosaccharides, including selectively sulfated *N*-glycans. The compounds and several other reference derivatives were used to construct a microarray that was probed for binding by several plant lectins, Siglec proteins, and hemagglutinins of influenza viruses. It was found that not only the sulfation pattern but also the presentation of epitopes as part of an *O*- or *N*-glycan determines binding properties.

## Introduction

**Figure 1:**
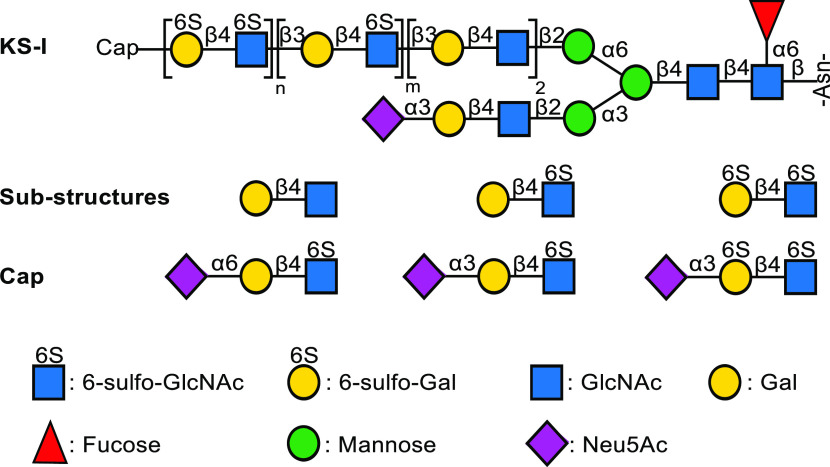
Structures of keratan sulfate oligosaccharides, including substructures of the repeating LacNAc chain and structures of capping epitopes.

Keratan sulfates (KS) are *N*- and *O*-linked glycans that occur in the extracellular matrix of many tissue types, where they can interact with a multitude of proteins, thereby controlling physiological and disease processes.^1−4^ One of the antennae of these *N*- and *O*-glycans is composed of poly-*N*-acetyl-lactosamine (poly-LacNAc) that can be modified by sulfation at the C-6 positions of *N*-acetyl-glucosamine (GlcNAc) and galactose (Gal) (Figure 1). Certain classes of KS can be further modified by 1,3-linked fucosides. KS has a modular architecture, and its backbone is composed of differently sulfated and fucosylated LacNAc moieties, and the termini can additionally be modified by 2,3- and 2,6-linked sialic acids.

The sulfated poly-LacNAc moieties of KS are assembled by a collection of glycosyltransferases and sulfotransferases.^5^ The consecutive action of β(1,3)-*N*-acetylglucosaminyltransferases (B3GnT) and β(1,4)-galactosyltransferases (B4GalT) results in the formation of the LacNAc backbone of KS. During the assembly of this chain, C-6 hydroxyls of terminal GlcNAc moieties can be sulfated by GlcNAc-6-*O*-sulfotransferases 2 and 6 (CHST2 and 6).^6−11^ The enzyme B4GalT4 can attach a 1,4-linked galactoside to 6-sulfo-GlcNAc residues, whereas B4GalT1 and B4GalT7 can extend unmodified GlcNAc residues. After assembly of the LacNAc chain, C-6 hydroxyls of galactosides can be sulfated by keratan sulfate galactose 6-sulfotransferase (KSGal6ST or CHST1) or chondroitin sulfotransferase-1 (C6ST1).^12,13^ β-Galactoside α-2,3-sialyltransferase 4 (ST3Gal4) and β-galactoside-α-2,6-sialyltransferase 1 (ST6Gal1) can install terminal 2,3- and 2,6-linked sialosides.

The biosynthesis of KS has mainly been examined by labeling studies of various oligosaccharides using ^35^S PAPS. It was found that only oligosaccharides having a GlcNAc moiety at the nonreducing end could be labeled when treated with CHST2.^8,14,15^ Furthermore, it was discovered that the combined use of B3GnT7 and B4GalT4 produced short oligosaccharides, whereas coincubation with CHST2 and PAPS produced extended sulfated LacNAc structures.^16^ Further labeling studies using ^35^S PAPS indicated that CHST1 modifies the internal galactosides of oligo-LacNAc derivatives and that the presence of sialic acid and GlcNAc6S enhances the incorporation.^12^ These observations indicate that the KS biosynthetic enzymes cooperate to create specific epitopes. The intricate details of the biosynthetic pathway are, however, not understood, and furthermore, a lack of well-defined KS structures has made it difficult to uncover the molecular basis by which it regulates biological processes.

Structural heterogeneity makes it challenging to obtain well-defined KS oligosaccharides from natural sources. Chemical approaches have been reported for the preparation of sulfated LacNAc derivatives; however, due to the need to perform time-consuming and demanding protecting manipulations and glycosylations, it has resulted only in relatively small structural motifs such as di- and tetrasaccharides.^17−21^ Sulfated LacNAc derivatives have been chemically synthesized and further modified by fucosylation and sialylation.^22^ In an interesting approach, chemically synthesized oxazolines were linked together by trans-glycosylation using a mutant form of keratanase II from *Bacillus* to give several oligosaccharides. The scope of this approach is restricted, however, due to a limited substrate tolerance and the fact that it cannot provide compounds larger than a hexasaccharide.^23,24^

Herein, we describe a chemo-enzymatic approach to prepare well-defined KS oligosaccharides by exploiting the known and newly discovered substrate specificities of relevant sulfotransferases. The premise of the approach is that recombinant CHST2^25^ only sulfates terminal GlcNAc moieties to give GlcNAc6S that can be galactosylated by B4GalT4 to provide Gal(β1,4)GlcNAc6S. Furthermore, CHST1 can modify the internal galactosides of a poly-LacNAc chain; however, it was found that a GlcNAc6S residue greatly increases the reactivity of the neighboring and internal galactosides. The presence of a 2,3-linked sialoside further modulates the site of modification by CHST1, and a galactoside flanked by 2,3-Neu5Ac and GlcNAc6S is preferentially sulfated over other galactosyl residues. The substrate specificities of CHST1 and 2 were exploited for the preparation of a panel of KS oligosaccharides, including selectively sulfated *N*-glycans. The library of oligosaccharides and several other reference compounds were used to construct a microarray that was probed with lectins, glycan-binding proteins, and hemagglutinins of influenza A viruses. It was found that sulfation can modulate recognition and can either be tolerated, enhance, or reduce binding. Furthermore, we discovered that the presentation of sulfated epitopes in the context of *N*- and *O*-glycans can greatly impact binding.

## Results and Discussion

**Scheme 1:**
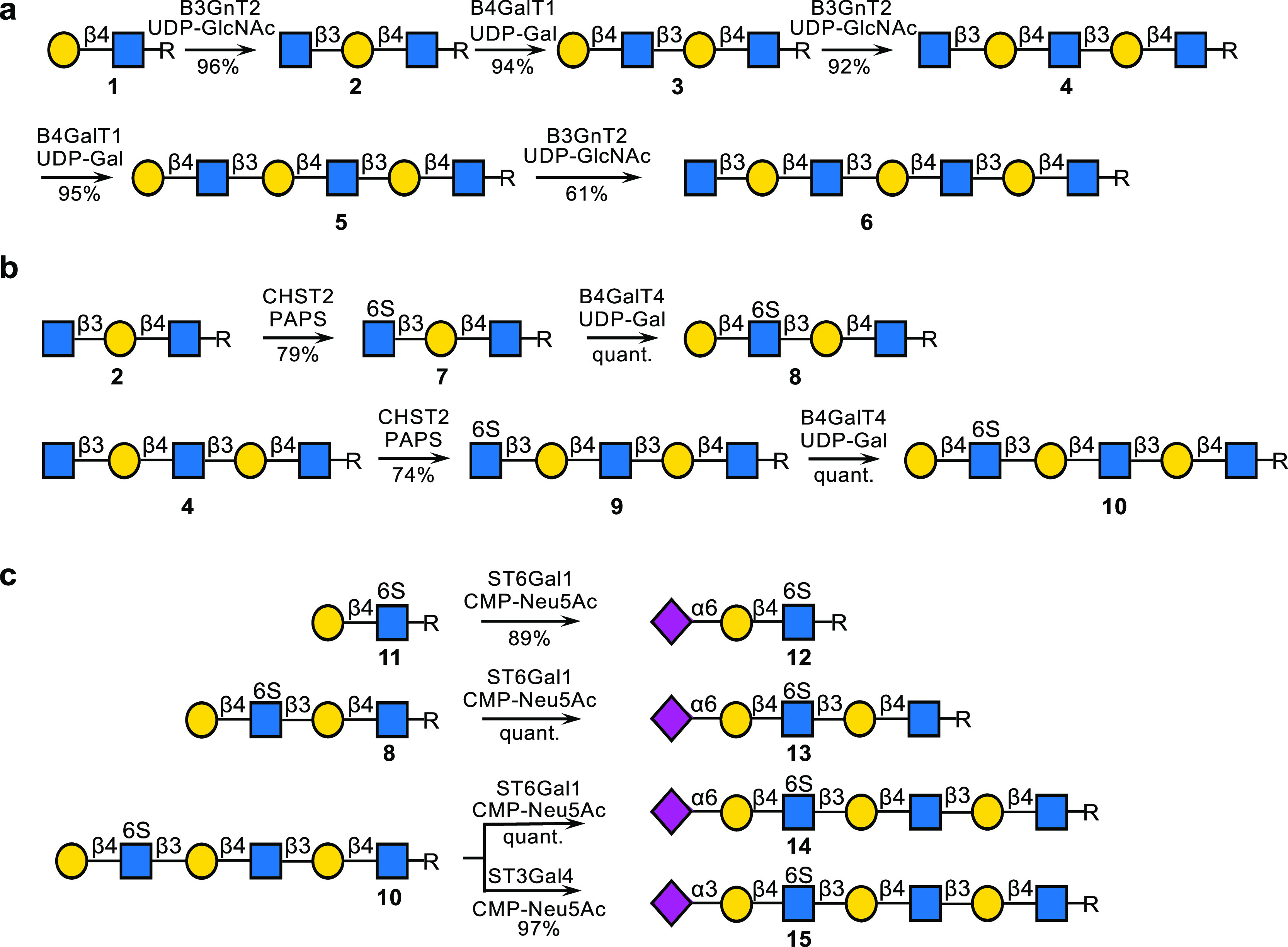
Enzymatic Synthesis of Linear Keratan Sulfate Oligosaccharides; (a) Assembly of polyLacNAc Backbone; (b) Elongation of GlcNAc6S; (c) Cap Introduction of Keratan Sulfate R = O(CH_2_)_5_NHCbz.

**Scheme 2:**
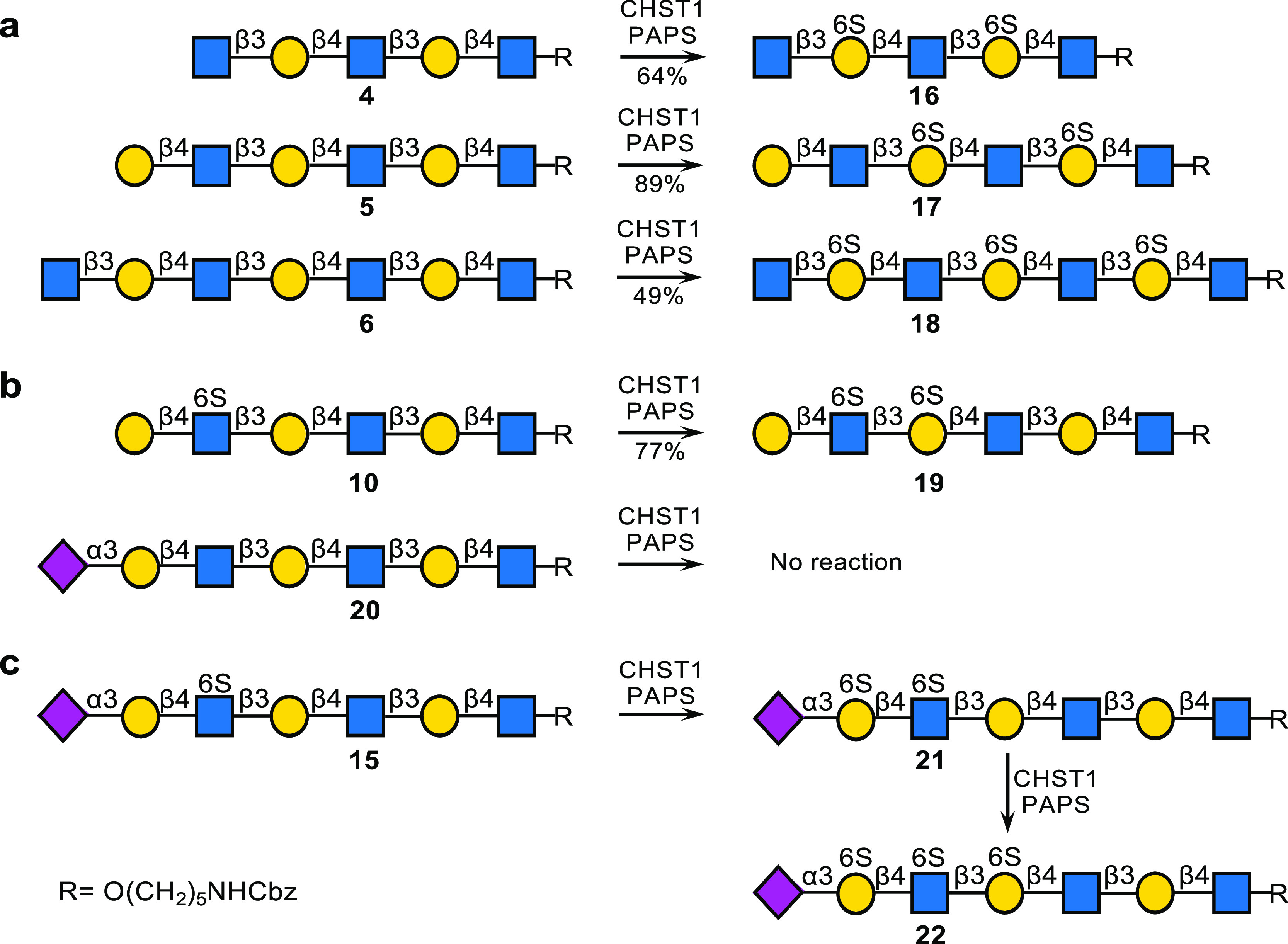
Using Well-Defined Oligosaccharides to Examine the Substrate Specificity of CHST1; R = O(CH_2_)_5_NHCbz; (a) Sulfation of Oligo-LacNAc Derivatives; (b) Modification by CHST1 of Sulfated or Sialylated Derivatives; (c) CHST1 Modification of Compounds Having a Sialoside and Sulfate

LacNAc derivatives **4**–**6** were prepared to examine the substrate specificities of CHST1 (Scheme 1a). In addition, compounds **11**, **8**, and **10** were used to examine the influence of GlcNAc6S on sialylation. Finally, compounds **10**, **20**, and **15** were employed to examine in what way sulfation at the C-6 position of GlcNAc and 2,3-sialylation influence the regioselectivity of CHST1 (Scheme 2b,c). We exploited the ecto-domains of recombinant B4GalT1 and B3GnT2^25^ in combination with UDP-Gal and UDP-GlcNAc to assemble the unmodified oligo-LacNAc moieties **4**–**6** (Scheme 1a) starting from chemically synthesized LacNAc derivative **1**. The latter compound has a benzyloxycarbonyl (Cbz)-protected aminopentyl spacer at the anomeric center, which, after deprotection, facilitates glycan microarray construction. Thus, treatment of **1** with B3GnT2 and UDP-GlcNAc gave **2**, which was further treated with B4GalT1 in the presence of UDP-Gal to provide **3**. Another cycle of enzymatic modification by B3GnT2 and B4GalT1 gave access to **5** and **6**. The compounds were purified by size exclusion column chromatography over Bio-Gel P2 or P6 and fully characterized by homo- and heteronuclear two-dimensional NMR experiments and by LC–MS. The compounds were prepared on scales ranging from 3 to 10 mg. Reaction mixtures were incubated for 18 h and performed at concentrations of 2–5 mM.

Next, attention was focused on the sulfation and further elongation of compounds **2** and **4**. Treatment of these compounds with CHST2 in the presence of PAPS resulted in the facile formation of **7** and **9** in quantities of 7.1 and 2.4 mg, respectively. Detailed NMR analysis confirmed that sulfation had occurred at the terminal GlcNAc moiety. For example, the 1D ^1^H NMR and 2D ^13^C–^1^H HSQC spectra of compound **7** (see Supporting Information) made it possible to assign all proton and carbon signals. The 6-carbon of the terminal GlcNAc moiety had shifted downfield (δ 60.2 → δ 67.2), and the corresponding protons also exhibited a chemical shift difference (H6a δ 3.98 → 4.34, H6b 3.83 → 4.23), which confirmed the regioselectivity of sulfation. The inter-residue connectivity was confirmed by a NOESY spectrum, which showed interactions of H-1 of GlcNAc-C with H-3 Gal-B and H-1 of Gal-B with H-4 of GlcNAc-A in accordance with C(1→3)B and B(1→4)A linkages, respectively.

Surprisingly, during the preparation of **7**, a trace amount of disulfated product was detected by LC–MS, which could readily be removed by diethylaminoethyl (DEAE) ion exchange column chromatography. The main product was, however, terminally sulfated, confirming the substrate specificity of CHST2 (Scheme S1 in Supporting Information). Compounds **7** and **9** could readily be galactosylated by using B4GalT4 in the presence of UDP-Gal to provide compounds **8** and **10**, respectively. Other galactosyltransferases were also examined, and for example, B4GalT1 was able to modify **7** and **9**, but the rate of transfer was very slow, and the reaction could not be driven to completion. HpGalT^26^ was able to fully modify **7** and **9**, but the rate of transfer was substantially slower than for B4GalT4. The presence of a sulfate at the terminal LacNAc moieties of compounds **11**, **8**, and **10** did not impede sialylation by ST3Gal4 and ST6Gal1, and compounds **12**–**15** could readily be prepared in quantities ranging from 1 to 3 mg.

Compounds **4**–**6** and **10**, **20**, and **15** were used to explore the substrate specificities of CHST1 (Scheme 2). First, the linear LacNAc substrates **4**, **5**, and **6** were treated with CHST1 in the presence of PAPS (1.5 equiv per galactose residue in the galactoside), and the progress of the reactions was monitored by LC–MS. Although the reactions were slow, all galactosyl moieties of compounds **4** and **6** were sulfated at C-6 to provide, after purification by size exclusion column (P6) and ion exclusion column (DEAE) chromatography, the sulfates **16** and **18**, respectively. On the other hand, hexasaccharide **5** was only sulfated at the internal galactosides to give disulfate **17** in high yield.

To examine the influence of 6-O-sulfation of GlcNAc, compound **10** was incubated with CHST1 in the presence of PAPS, which resulted in the formation of a single compound (Scheme 2b). Detailed NMR analysis demonstrated that sulfation had occurred at the −1 site to give compound **19** in a quantity of 0.4 mg. For example, the 1D ^1^H NMR and 2D ^13^C–^1^H HSQC spectra of compound **19** (Supporting Information) made it possible to assign all proton and carbon signals. The 6-carbon of the internal sulfated galactose moiety had substantially shifted downfield (δ 61.3 → δ 67.9), and the corresponding protons also exhibited a chemical shift difference (H6 δ 3.76 → 4.20), which confirmed the regioselectivity of sulfation. The inter-residue connectivity was confirmed by a NOESY spectrum. The inter-residue connectivities Gal-F H-1, GlcNAc-E H-4, GlcNAc-E H-1, Gal-D H-3, Gal-D H-1, GlcNAc-C H-4, GlcNAc-C H-1, Gal-B H-3, and Gal-B H-1, GlcNAc-A H-4 are in accordance with F(1→4)E, E(1→3)D, D(1→4)C, C(1→3)B, and B(1→4)A linkages, respectively. Surprisingly, sialoside **20** was not sulfated by CHST1, indicating that sialic acid deactivates all of the galactosides from modification (Scheme 2b). It implies that CHST1 senses the modifications at the nonreducing terminus of the polymer, impacting the modification of internal sites. A combination of GlcNAc6S and a 2,3-linked sialoside at a terminal LacNAc moiety, as in compound **15** (Scheme 2c), changed the site of sulfation, and in this case, treatment with CHST1 in the presence of PAPS (1.6 equiv) resulted in the formation of **21** as the major product, which has a sulfate at the Gal moiety that is flanked by the sialoside and GlcNAc6S. A small amount of additionally sulfated **22** was formed, which could easily be removed by DEAE ion exchange column chromatography. Further incubation of **21** in the presence of PAPS for a prolonged period of time resulted in complete sulfation of the Gal moiety at the −1 site, providing compound **22**. These results highlight a complex interplay between sialylation and sulfation to introduce terminal epitopes, and only the presence of a 2,3-linked sialoside and GlcNAc6S activates the C-6 of the galactose of the terminal LacNAc moiety for sulfation by CHST1. The latter sulfotransferase can also modify the galactoside at the −1 site, albeit at a lower rate of modification.

**Scheme 3:**
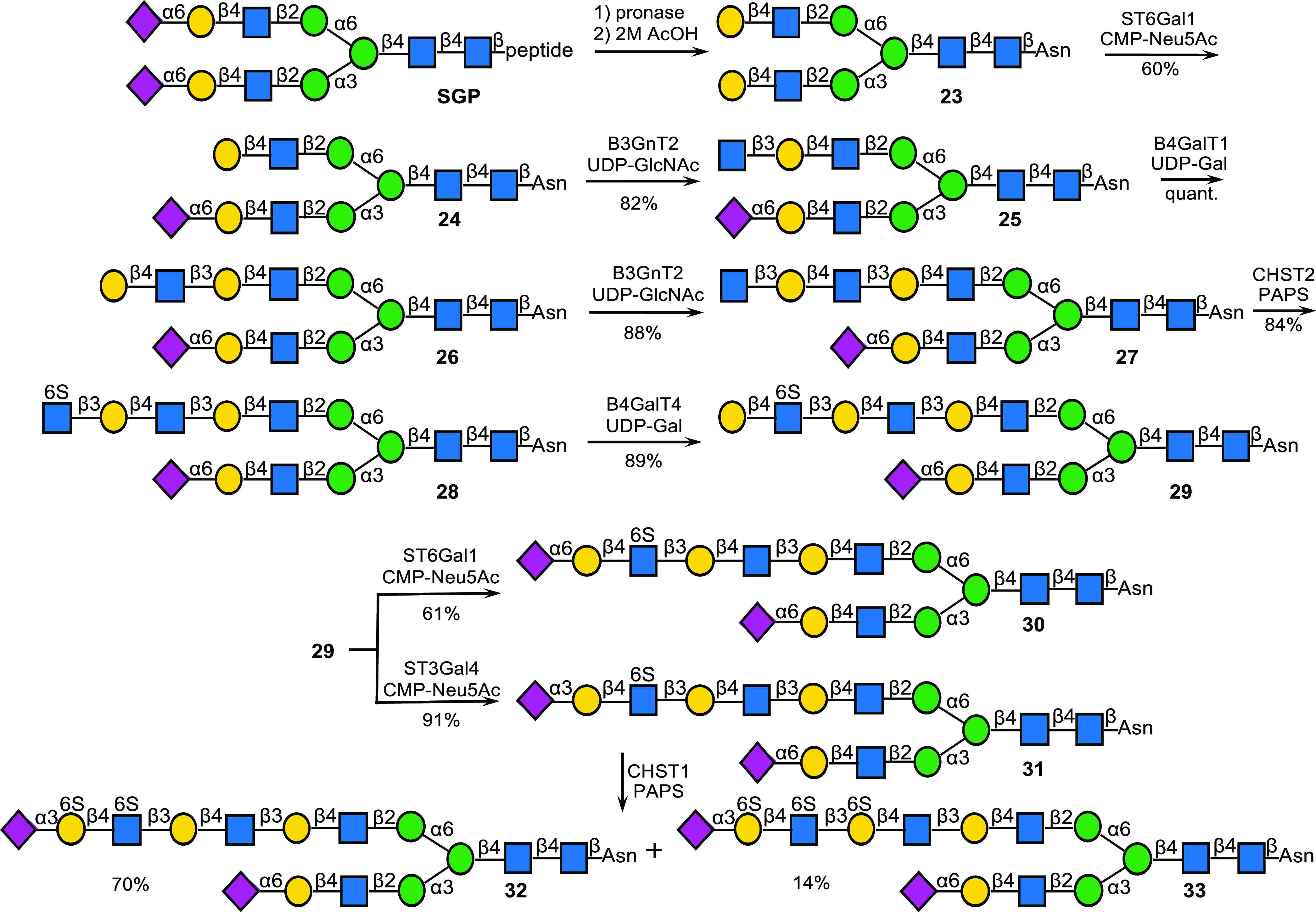
Chemoenzymatic Synthesis of KS-I Oligosaccharides by Enzymatic Modification of an *N*-Linked Glycan Obtained from Egg Yolk Powder and Exploitation of the Regioselectivities of CHST1 and CHST2

The newly discovered selectivities of CHST1 and CHST2 were exploited to enzymatically prepare several *N*-linked glycans. Sialoglycopeptide (SGP, Scheme 3), which can be isolated in multigram quantities from egg yolk powder, was converted into biantennary glycosyl asparagine **23** by subsequent Pronase treatment to remove the peptide moiety and hydrolysis of the sialosides with 2 M AcOH.^27,28^ Next, ST6Gal1, which has a preference for the MGAT1 arm, was used to desymmetrize **23** to give monosialoside **24**.^29^ The terminal galactoside of **24** was extended by subsequent modification by B3GNT2 (→**25**), B4GalT1 (→**26**), and B3GNT2 to provide **27**. As expected, the terminal GlcNAc moiety of **27** could selectively be sulfated by CHST2 in the presence of PAPS to give **28**, which was further treated with B4GalT4 and UDP-Gal to provide compound **29**. As expected, the terminal Gal of **29** could readily be sialylated by ST6Gal1 or ST3Gal4, resulting in the formation of compounds **30** and **31**, respectively. Finally, **31** was subjected to CHST1 and PAPS (1.5 equiv), which gave **32** as the major compound and a small amount of **33** in quantities of 1.0 and 0.2 mg. The two compounds could readily be separated by DEAE ion exchange column chromatography and were fully characterized by NMR and LC–MS.

The Cbz protecting group of the enzymatically prepared compounds was removed by hydrogenation over Pd(OH)_2_, and after purification by P6 size-exclusion column chromatography using 50 mM ammonium bicarbonate as eluent, the corresponding aminopentenyl derivatives were obtained (**11** → **A**, **8** → **B**, **10** → **C**, **12** → **G**, **13** → **H**, **14** → **I**, **20** → **J**, **15** → **K**, **21** → **L**, **31** → **N**, **32** → **O**, and **33** → **P**).

**Figure 2:**
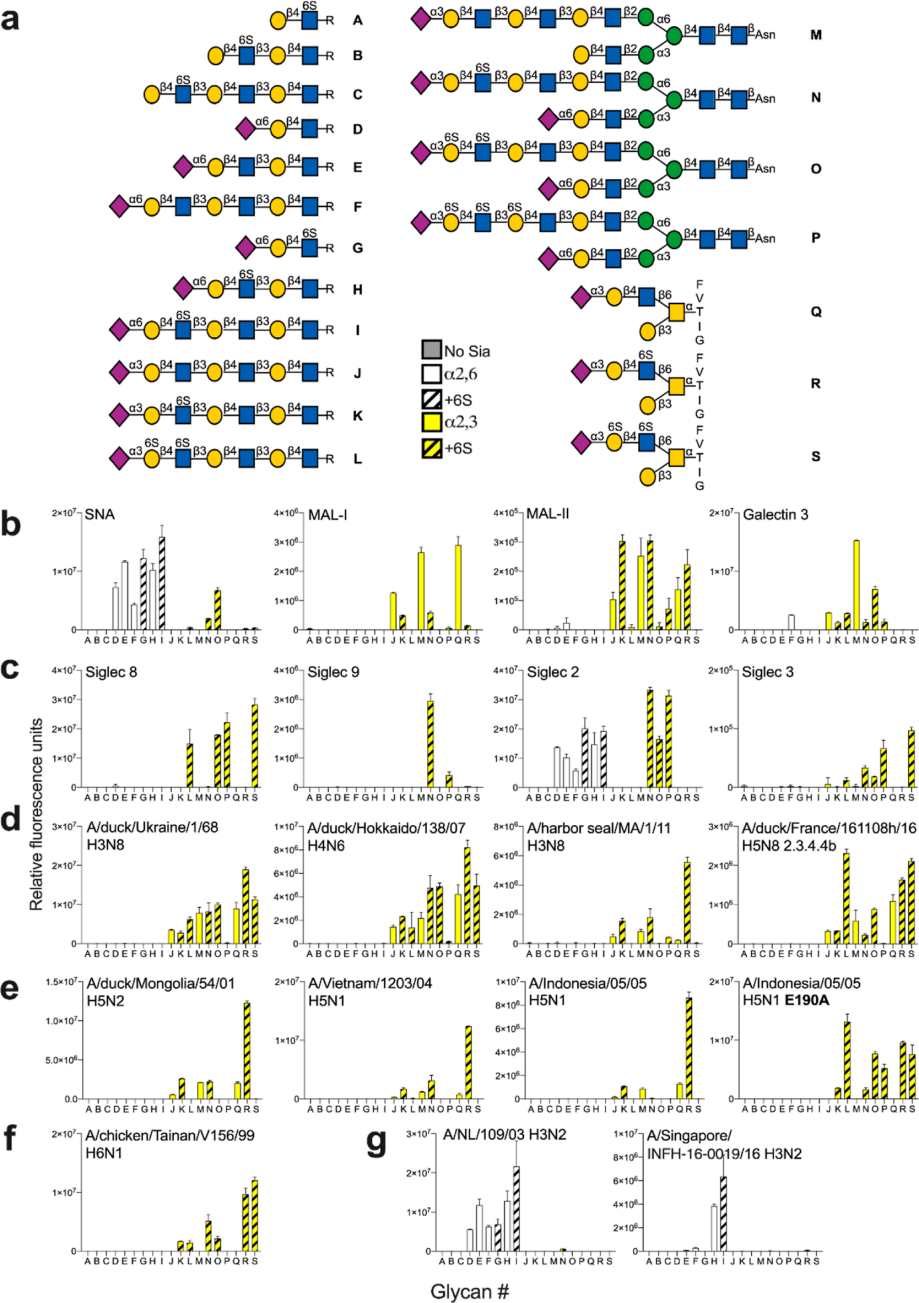
Probing glycan binding properties of lectins, glycan binding proteins, and influenza A hemagglutinins. (a) Collection of glycans printed on succinimide-reactive microarray slides. Glycan binding data of (b) lectins, (c) Siglecs, and (d–g) HAs of various influenza viruses. Bars represent the background-subtracted average relative fluorescence units (RFU) of four replicates ±SD.

The newly synthesized glycans and several reference compounds, which include several *O*-glycans presenting relevant epitopes (**Q**-**S**), were employed to construct a glycan microarray. The linear derivatives have an anomeric aminopentyl and the *N*-glycans an asparagine moiety, which facilitated printing on amine-reactive *N*-hydroxysuccinimide (NHS)-activated glass slides to give a glycan microarray (Figure 2a). The array was probed by several plant lectins and galectin-3 (Gal-3) (Figure 2b), sialic acid-binding immunoglobulin-type lectins (Siglecs) (Figure 2c), and recombinant hemagglutinins (HAs) of animal and human influenza viruses (Figure 2d–g). As expected, MAL-I and MAL-II bound 2,3-sialylated LacNAc-containing structures (**J**, **M**, and **Q**). In the case of MAL-1, sulfation at GlcNAc (**K**, **N**, and **R**) substantially reduced the responsiveness, whereas for MAL-II it was tolerated. Further sulfation at Gal (**L**, **O**, and **S**) was not allowed by these lectins. SNA recognizes 2,6-linked sialosides, and as expected, compounds (**D**-**I**) having such a structural element were well recognized by this lectin, and it appears that sulfation of GlcNAc, as in compounds **G**, **H**, and **I**, did not impede binding. Compounds **N**–**P** gave relatively low responses, indicating that the 2,6-sialoside at the MGAT1 arm is not well recognized by this lectin, possibly through interference by the other arm.

Siglecs are receptors expressed by cells of the immune system that can bind specific sialic acid-containing glycoconjugates, thereby modulating immune responses.^30^ The sialic acid–Siglec axis plays an important role in the balance between self- and nonself and is disturbed in diseases such as cancer, autoimmunity, and allergy.^31^ Genetic engineering of cells has indicated that sulfation can regulate Siglec binding; however, the precise molecular mechanisms of this regulation are not well understood.^32,33^

Histological studies have shown that airway tissues express high-molecular-weight ligands for Siglec-8 and 9.^34^ Treatment of these tissues with keratanase-I abolished the binding of recombinant Siglec-8. There is evidence to support the idea that in inflamed tissue, human eosinophils and mast cells, which express Siglec-8, bind to sialoglycans to resolve inflammation and limit tissue damage.^34^ Previous glycan array studies indicated that Siglec-8 binds to glycans having a terminal Neu5Acα2,3(6-sulfo)-Gal moiety.^35^ The enzymatic studies described here have indicated that sulfation of Gal most likely occurs in the context of a neighboring (6-sulfo)-GlcNAc moiety, and thus, these ligands are the most likely natural candidates for Siglec-8. Indeed, when the microarray was probed with recombinant Siglec-8, binding was only observed to compounds **L**, **O**, **P**, and **S**, which have a sialoside 2,3-linked to a LacNAc moiety sulfated at Gal and GlcNAc (Figure 2c). It appears that presentation in the context of an *N*- or *O*-glycan does not modulate binding, and compound **O** and **S** showed similar responsiveness. Siglec-8 tolerates sulfation of the subsequent Gal, as in compound **P**, which is in agreement with the finding that this Siglec binds to KS.^35^ Siglec-9 exhibited a different binding pattern and was preferentially bound to compound **N,** which has a Neu5Acα2,3Gal β1,4GlcNAc6S moiety presented in the context of an *N*-glycan (Figure 2c). When this epitope was part of an *O*-glycan, as in compound **R**, the level of binding was diminished. Siglec-2 (CD22) is expressed by B-cells and recognizes 2,6-linked sialoglycans. The array data showed that it tolerates sulfation at the GlcNAc moiety (**D** vs **G**, **E** vs **H**, **F** vs **I**). Siglec-3 (CD33) controls the activation of microglial cells; however, in Alzheimer disease, it is overactivated due to the presence of amyloid and tau proteins. Siglec-3 showed a binding pattern that is similar to that of Siglec-8 (Figure 2c). Finally, we investigated the binding properties of Gal-3, which prefers extended LacNAc moieties, and as expected, compound **M** was well recognized, whereas sulfation of the extended LacNAc chain resulted in substantially reduced binding (Figure 2b).

The expression of KS in airway tissues makes these biomolecules potential candidates as receptors for respiratory viruses. Other GAGs, such as heparan sulfate, have been implicated in viral infections;^36^ however, the role of KS in such processes has received little attention. Hemagglutinin (HA) of influenza A virus binds to the sialoglycans of the host for cell entry. Avian viruses preferentially bind 2,3-linked sialic acids, whereas human viruses recognize α2,6-linked sialic acids. Several human and avian viral HAs can bind to sialoglycans modified by sulfates or fucosides, and such binding preferences may represent species barriers.^37−40^ We used the array to examine the binding selectivity of several recombinant HAs that are derived from viruses that infect different host species, such as H3N8 and H5Nx viruses. Additionally, HAs derived from other subtypes that are mostly maintained in avian species were also examined.

Recombinant HAs derived from duck H3N8 and H4N6 viruses bound 2,3-linked sialoside and tolerated sulfation at Gal as well as GlcNAc, and the sulfates had relatively little impact on the responsiveness (Figure 2d). Further sulfation of Gal, as in compound **P**, was not tolerated. Another duck virus (A/duck/France/161108h/16, H5N8), which is representative of current epizootic outbreaks,^41^ exhibited a similar promiscuous binding behavior (Figure 2d). HA derived from a harbor seal H3N8 virus showed a different binding pattern and allowed only sulfation in GlcNAc (Figure 2d). Presentation of the Neu5Ac2,3-Gal-GlcNAc6S epitope in the context of an *O*-glycan resulted in the strongest binding (**Q** vs **R**).

Several H5 proteins (A/duck/Mongolia, A/Indonesia, and A/Vietnam) also showed a strong preference for an *O*-glycan presenting the Neu5Ac2,3-Gal-GlcNAc6S epitope (**R**) (Figure 2e). In a previous study, we analyzed amino acid mutations that occurred during a human infection, one of which diminished binding to sialylated LacNAc (A/Vietnam E190A).^42^ Here, we observed that this mutant HA exclusively binds receptors that include a 6-sulfated GlcNAc.

An HA from a chicken H6N1 virus had an obligatory requirement for sulfation of GlcNAc (**K**, **N**, and **R**) and tolerated further sulfation of Gal (**L**, **O**, and **S**) (Figure 2f).^43^ The highest responsiveness was observed when the sulfated epitope was presented as part of an *O*-glycan. We also examined the receptor specificities of two human H3N2 viruses (Figure 2g). As expected, these viruses are only bound to 2,6-linked sialosides. The more recent A/H3N2/Singapore virus required the presentation of the 2,6-linked sialoside at an extended LacNAc moiety (**H**), which agrees with previous observations.^44^ Interestingly, both viruses tolerated a GlcNAc6S moiety (**F** vs **G** and **H** vs **I**). Collectively, the microarray screening indicates that not only sialic linkage type but also sulfation and presentation of an epitope as part of an *N*- or *O*-glycan can modulate receptor specificity. In the case of *N*-linked structures, the terminal sialyl LacNAc moiety is attached to a subsequent β1,3-linked LacNAc moiety, whereas in the case of *O*-glycans, it is linked to a β1,6-linked GalNAc residue. It is possible that larger structures than sulfated sialyl LacNAc moieties are recognized, explaining the difference in binding between *O*- and *N*-linked glycans. It is also possible that the glycan moiety underlying the minimal epitope has unfavorable interactions with the protein or causes differences in conformational properties. Further structural studies are required to provide molecular insight into how the complexity of KS oligosaccharides influences recognition.

## Conclusions

The results presented here show that GlcNAc6S moieties and 2,3-sialylation determine the preferred sites of sulfation by CHST1. In combination with the regioselectivity of CHST2, which only modifies terminal GlcNAc moieties, it was possible to prepare a range of well-defined KS oligosaccharides. The compounds and several reference derivatives were printed as a glycan microarray, which uncovered the binding selectivities of a range of lectins, glycan-binding proteins, and HAs from influenza A viruses. It was found that sulfation can greatly influence recognition and can either be tolerated, reduce, or increase binding. Modification of Gal and GlcNAc exerts differential effects, which are further influenced by the presentation of a sulfated epitope as part of an *N*- or *O*-glycan. The established structure-binding relationships highlight that glycan complexity can modulate protein binding properties. Future efforts will focus on the preparation of a broader range of KS oligosaccharides to examine the effects of sulfation on protein binding.

## Methods

### General Procedure for the Installation of β1,3-GlcNAc Using B3GnT2

Glycosyl acceptor (1 equiv) and UDP-GlcNAc (1.5 equiv) were dissolved to provide a final acceptor concentration of 2–5 mM in a HEPES buffered solution (50 mM, pH 7.3) containing KCl (25 mM), MgCl_2_ (2 mM) and DTT (1 mM). Calf intestine alkaline phosphatase (CIAP, 1% total volume, 1 kU/mL) and B3GnT2 (1% w/w relative to acceptor substrate) were added, and the reaction mixture was incubated overnight at 37 °C with gentle shaking. The progress of the reaction was monitored by MALDI-TOF MS or ESI-TOF MS, and if starting material remained after 18 h another portion of B3GnT2 was added until no starting material could be detected. The reaction mixture was centrifuged over a Nanosep Omega ultrafiltration device (10 kDa MWCO) to remove proteins, and the filtrate was lyophilized. The residue was applied to P2 or P6 size-exclusion column chromatography using Milli-Q water as eluent, providing the desired product. High-performance liquid chromatography (HPLC) using a HILIC column (see Materials) was employed when impurities were detected.

### General Procedure for the Installation of β1,4-Gal Using B4GalT1

Glycosyl acceptor (1 equiv) and UDP-Gal (1.5 equiv per Gal to be added) were dissolved to provide an acceptor concentration of 2–5 mM in a Tris buffered solution (100 mM, pH 7.5) containing MnCl_2_ (10 mM) and BSA (1% total volume). CIAP (1% volume total) and B4GalT1 (1% w/w relative to acceptor substrate) were added, and the reaction mixture was incubated overnight at 37 °C with gentle shaking. Progress of the reaction was monitored by MALDI-TOF MS or ESI-TOF MS, and if starting material remained after 18 h, another portion of B4GalT1 was added until no starting material could be detected. The reaction mixture was centrifuged over a Nanosep Omega ultrafiltration device (10 kDa MWCO) to remove proteins, and the filtrate was lyophilized. The residue was applied to P2 or P6 size-exclusion column chromatography using Milli-Q water as the eluent, providing the desired product. HPLC using the HILIC column (see Materials) was employed when impurities were detected.

### General Procedure for the Installation of β1,4-Gal Using B4GalT4

Glycosyl acceptor (1 equiv) and UDP-Gal (1.5 equiv per Gal to be added) were dissolved to provide an acceptor concentration of 2–5 mM in a Tris buffered solution (100 mM, pH 7.5) containing MnCl_2_ (10 mM) and BSA (1% total volume). CIAP (1% volume total) and B4GalT4 (1% w/w relative to acceptor substrate) were added, and the reaction mixture was incubated overnight at 37 °C with gentle shaking. Reaction progress was monitored by MALDI-TOF MS or ESI-TOF MS, and if the starting material remained after 18 h another portion of B4GalT4 was added until no starting material could be detected. The reaction mixture was centrifuged over a Nanosep Omega ultrafiltration device (10 kDa MWCO) to remove reaction proteins, and the filtrate was lyophilized. The residue was applied to P2 or P6 size-exclusion column chromatography using Milli-Q water as eluent, which provided the desired product. HPLC using a HILIC column (see Materials) was employed when impurities were remaining.

### General Procedure for the Installation of α2,3-Neu5Ac Using ST3Gal4

Glycosyl acceptor (1 equiv) and CMP-Neu5Ac (1.5 equiv) were dissolved at a final acceptor concentration of 2–5 mM in a HEPES-buffered solution (50 mM, pH 7.2) containing BSA (1% total volume). CIAP (1% volume total) and ST3Gal4 (1% w/w relative to acceptor substrate) were added, and the reaction mixture was incubated overnight at 37 °C with gentle shaking. Progress of the reaction was monitored by ESI-TOF MS, and if starting material remained after 18 h, another portion of ST3Gal4 was added until no starting material could be detected. The reaction mixture was centrifuged over a Nanosep Omega ultrafiltration device (10 kDa MWCO) to remove proteins, and the filtrate was lyophilized. The residue was applied to P2 or P6 size-exclusion column chromatography using Milli-Q water as eluent to provide the desired product. HPLC using a HILIC column (see Materials) was employed when impurities were detected.

### General Procedure for the Selective Installation of Terminal α2,6-Neu5Ac Using ST6Gal1

Glycosyl acceptor (1 equiv) and CMP-Neu5Ac (1.1 equiv) were dissolved at a final acceptor concentration of 2–5 mM in a HEPES-buffered solution (100 mM, pH 7.5) containing BSA (1% volume total). CIAP (1% volume total) and ST6GAL1 (1% w/w relative to acceptor substrate) were added, and the reaction mixture was incubated overnight at 37 °C with gentle shaking. The reaction mixture was centrifuged over a Nanosep Omega ultrafiltration device (10 kDa MWCO) to remove reaction proteins, and the filtrate was lyophilized. The residue was applied to P2 or P6 size-exclusion column chromatography using Milli-Q water as the eluent to provide the desired product. HPLC using the HILIC column (see Materials) was employed when impurities were detected (see Compound 5 for further details).

### General Procedure for the 6-*O*-Sulfate Installation of Terminal GlcNAc Using CHST2

Glycosyl acceptor (1 equiv) and PAPS (1.6 equiv) were dissolved at a final acceptor concentration of 2–5 mM in a Tris buffered solution (100 mM, pH 7.5) containing MgCl_2_ (10 mM). CHST2 (10–20% w/w relative to acceptor substrate) was added, and the reaction mixture was incubated overnight at 37 °C with gentle shaking. The reaction mixture was centrifuged over a Nanosep Omega ultrafiltration device (10 kDa MWCO) to remove reaction proteins, and the filtrate was lyophilized. The residue was applied to P2 or P6 size-exclusion column chromatography using NH_4_HCO_3_ buffer (50 mM) as the eluent to provide the desired product. HPLC using a HILIC column (see Materials) or the DEAE ion exchange column was employed when impurities were detected.

### General Procedure for the 6-*O*-Sulfate Installation of Internal Galactose Using CHST1

Glycosyl acceptor (1 equiv) and PAPS (1.6 equiv per galactose) were dissolved at a final acceptor concentration of 2–5 mM in a Tris buffered solution (100 mM, pH 7.5) containing MgCl_2_ (10 mM). CHST1 (10% w/w relative to the acceptor substrate) was added, and the reaction mixture was incubated overnight at 37 °C with gentle shaking. The reaction mixture was centrifuged over a Nanosep Omega ultrafiltration device (10 kDa MWCO) to remove reaction proteins, and the filtrate was lyophilized. The residue was applied to P2 or P6 size-exclusion column chromatography using NH_4_HCO_3_ buffer (50 mM) as the eluent to provide the desired product. HPLC using a HILIC column (see Materials) was employed when impurities were detected.

### Procedure for Rate-Controlled Synthesis of Compounds **32** and **33** Using CHST1

Glycosyl acceptor **31** (1 equiv) and PAPS (1 equiv) were dissolved at a final acceptor concentration of 2 mM in a Tris buffered solution (100 mM, pH 7.5) containing MgCl_2_ (10 mM). CHST1 (10% w/w relative to the acceptor substrate) was added, and the reaction mixture was incubated overnight at 37 °C with gentle shaking. Another portion of PAPS (0.5 equiv) was added, followed by incubation at 37 °C with gentle shaking. The progress of the reaction was monitored by ESI-TOF MS until **31** was not further consumed. The reaction mixture was centrifuged over a Nanosep Omega ultrafiltration device (10 kDa MWCO) to remove proteins, and the filtrate was lyophilized. The residue was applied to P2 or P6 size-exclusion column chromatography using NH_4_HCO_3_ buffer (50 mM), as the eluent provided the desired product. HPLC using a HILIC column (see Materials) or DEAE ion exchange column was employed when the impurities were remaining.

### General Procedure for Hydrogenation of Cbz-Protecting Group Using Pd(OH)_2_

Palladium hydroxide on carbon (Degussa type, 20%, 1.5 times the weight of the starting material) was added to a solution of the starting material in H_2_O (0.1% AcOH as an additive). The mixture was placed under an atmosphere of hydrogen until ESI-LC-MS indicated completion of the reaction. The mixture was filtered through a spin filter, and the residue was washed with H_2_O. The filtrate was lyophilized to give the final product. P6 size-exclusion column chromatography was used for purification with 50 mM ammonium bicarbonate as the eluent. The fractions containing compounds were lyophilized to give the desired product as a white powder.

## Supporting Information Available

Compound characterization, experimental procedure for enzyme and protein expression, microarray screening, and copies of NMR spectra (PDF)
